# Rye Bread Defects: Analysis of Composition and Further Influence Factors as Determinants of Dry-Baking

**DOI:** 10.3390/foods9121900

**Published:** 2020-12-19

**Authors:** Marie Oest, Ute Bindrich, Alexander Voß, Heinz Kaiser, Sascha Rohn

**Affiliations:** 1 Institute of Food Chemistry, Hamburg School of Food Science, University of Hamburg, Grindelallee 117, 20146 Hamburg, Germany; marie.oest@chemie.uni-hamburg.de; 2 German Institute of Food Technologies (DIL) e. V., Prof.-von-Klitzing-Str. 7, 49610 Quakenbrueck, Germany; U.Bindrich@dil-ev.de; 3 Institute for Food and Environmental Research (ILU) e. V., Papendorfer Weg 3, 14806 Bad Belzig, Germany; alexander.voss@ilu-ev.de (A.V.); dr.heinz.kaiser@t-online.de (H.K.); 4 Department of Food Chemistry and Analysis, Institute of Food Technology and Food Chemistry, Technische Universität Berlin, TIB 4/3-1, Gustav-Meyer-Allee 25, 13355 Berlin, Germany

**Keywords:** rye, dry-baking, bread defects, nonstarch-polysaccharides, proteins

## Abstract

For decades, the evaluation of rye milling products have been aimed at detecting raw material defects that are linked to excessive enzyme activity. Those defects were indirectly characterized by the rheological methods of the dough or the final products. However, such methods do not sufficiently reflect the baking properties of all rye flours present on the market. A further problem is the continuing climate change, which affects compound composition in rye. So far, these bread defects can only be corrected by process engineering (e.g., extended dough resting). Therefore, it is necessary to characterize the main determinants of the quality defects prior to the baking process in order to predict baking quality and not waste raw material, energy, and time. In this study, it was found that the water accessibility of starch for gelatinization and its partial inhibition by certain components play a major role in baking quality. Specifically, high amounts of insoluble nonstarch-polysaccharides (NSPSs) and a hindered denaturation of proteins seem to be an indication and reason for poor baking quality. However, traditional quantitative analysis of the ingredients and properties of the rye milling products (e.g., falling number, protein content, amylographic data) does not allow any reliable conclusions about rye flour suitability for use as bread rye. It can be concluded that more complex compositional aspects (e.g., complexation of compounds) need to be characterized for future quality control of rye.

## 1. Introduction

Traditional rye bread is the term used for rye and rye-containing mixed bread, which, according to the official German food guidelines (“*Leitsätze des Deutschen Lebensmittelbuchs für Brot und Kleingebäck*”), consist of more than 50% of rye flour fractions and are usually produced with sourdough [1]. In the quality assessment of rye bread, defects such as lack of freshness, a nonjuicy crumb, deficiencies in chewing properties, cracks in the crumb, as well as loosening and volume defects are increasingly occurring, regardless of company/bakery size or processing practices [2]. For these unsatisfactorily baking results of flours with almost consistently high viscosity maxima in their amylograms, but no further detailed evidence to the cause, the term “dry-baking” has been established [2,3,4].

However, one overall reason for such a baking behavior seems to be the changing climate and further ecophysiological influence factors during the ripening and maturation of rye in the field [5,6]. Consequently, rye has been subjected to further optimization by breeding in order to adapt growth and yield to the changing conditions. As a cross-pollinator, rye is a cereal of which ecophysiological conditions have a major influence, besides genetic predisposition [7,8]. Current breeding and growth conditions, as well as climate change, result in an unfavorable (high) level of falling number, amylographic maxima, and starch gelatinization temperature [2,3,4].

In order to be classified as bread rye or feed rye, traditional parameters such as falling number and amylographic data are of decisive importance [9]. Moreover, the falling number is an important factor determining the price when purchasing rye [10]. As described above, the parameters for the characterization of rye flours underline the long-term trend of constant change with regard to falling number, as well as viscosity and temperature maximum in amylograms. However, these parameters do not lead to linear or consistent trends that allow us to draw direct conclusions explaining the phenomena of “dry-baking”.

The behavior of dough and the resulting bread quality are influenced by all components of rye. Therefore, individual components must be studied more comprehensively and specifically in order to potentially predict changed bread characteristics.

The most obvious compound class to be studied is the hemicelluloses, as rye dough is a suspension of insoluble solids (starch, insoluble proteins, shell components) in a viscous liquid phase, consisting mainly of nonstarch-polysaccharides (NSPSs). Hemicelluloses like arabinoxylan and beta-glucan are colloidally dissolved in water [11,12]. Thus, rye doughs differ fundamentally from wheat-based doughs, with the latter consisting of a continuous viscoelastic protein network based on typical gluten proteins [13,14]. The formation of such a protein network cannot be observed in rye dough. Rye doughs are considerably more plastic, and, furthermore, the amount of water added comparatively determines the processing properties of the doughs, in combination with the water-binding properties of further compounds.

In the context of water-binding capability, hemicelluloses, in particular pentosans and glucans, play a major role as they are able to absorb water up to 100 times their own mass [11,15,16,17], indicating a great potential for the formation of hydrogen bonds and, thus, high physicochemical reactivity. These properties are also responsible for the typical stickiness of rye doughs and are considered a prerequisite for the typical “juiciness” of the bread crumb [18].

With regard to sensory characteristics, the proportions of water -soluble and insoluble pentosans are also of importance. Insoluble pentosans possess a higher swelling capacity. However, with a higher content of soluble pentosans, more elastic and loose crumb characteristics can be achieved [19]. Stępniewska et al. (2018) reported that the quality of rye milling products could be predicted using a swelling curve test. Their study showed that crumb hardness could be negatively correlated with a change in the viscosity in the swelling curve test, whereas this change could be positively correlated with crumb moisture. The changes in the swelling curve are significantly influenced by a composition of soluble and insoluble pentosans [20].

Buksa et al. (2010) found that in bread made from wholemeal rye flour with low enzymatic activity, a higher bread volume is achieved when the pentosan fraction contains a large proportion of polymers that are extractable with water [21].

Besides NSPSs, rye also contains another group of polymers that are able to form functional network-selected proteins. The role of the proteins in rye dough formation has, so far, received little attention in terms of baking quality. Although the proteins principally form a wheat-gluten-like network, the interactions with hemicelluloses prevent the formation of typical gluten structures [22,23]. When taking a look at the literature, the dominant opinion is that the solubility of the proteins does not significantly influence the properties of rye dough or that there are no reliable findings regarding their function [11,24,25]. However, when considering the properties of doughs and baked goods, proteins, although not forming a protein network, must no longer be neglected with regard to affected rye properties.

In an attempt to investigate the functionality of the rye proteins in relation to bread quality, Beck et al. (2011) were able to create a rye dough structure that is similar to wheat dough in rye doughs by crosslinking the rye proteins with transglutaminase [26]. As a result, higher viscous and elastic proportions were achieved, indicating that the interactions between proteins and other ingredients and the accessibility of certain amino acid side chains play important roles in the formation of rye dough and, thus, the quality of rye baked goods.

With regard to dough formation and bread quality, it becomes apparent that overall composition is becoming more and more important for predicting the baking quality of rye. Torbica et al. (2019) showed that it is possible to predict specific bread volume, an important parameter for the evaluation of bread quality, by torque analysis using the Mixolab system [27]. This technique provides information about protein, hemicelluloses, starch, and other components and their interactions that influence dough and bread properties. The role of hemicelluloses and proteins and their potential interactions have not been sufficiently studied despite their important individual functional properties.

The problem behind this study has an intense history. In the last 10 years, rye harvests have been evaluated using several hundred rye samples and the use of a (modified) standard baking test [28]. However, this test is very laborious, time-consuming, and not applicable to the milling and bakery industry. This study aims at identifying simpler, easy-to-analyze parameters for rye quality evaluation and a certain predictability in the baking results. Consequently, this study focuses on a broader characterization of selected quality-defining parameters by using a selection of samples differing in baking quality. The traditional parameters being applied so far, such as the falling number and amylographic data, will be correlated with quantification results of soluble/insoluble hemicelluloses, proteins, and starch in order to evaluate the influence of these parameters on dough formation (explicitly, starch gelatinization) and the resulting bread quality.

## 2. Materials and Methods

### 2.1. Materials

α-Amylase (from Porcine pancreas, 500 kU/mL), L-(+)-arabinose, i-erythritol ≥ 99.0%, N,O-bis-(trimethylsilyl)-acetamide ≥ 99.0%, phloroglucinol ≥ 99,0%, sulfosalicylic acid ≥ 99.0%, and sorbitol ≥ 99.0% were all purchased from Sigma-Aldrich (St. Louis, MO, USA). Ethanol (absolute for analysis), 1,1,2-trichlorotrifluoroethane, Rhodamine R ~95%, and D-(+)-xylose were purchased from Merck KGaA (Darmstadt, Germany). Ulmer Boerol was purchased from CSM Deutschland GmbH (Bingen am Rhein, Germany). β-Glucan was purchased from Glucagel® (DKSH Management Ltd., Zurich, Switzerland). Dichloromethane ≥ 99.8%, sodium chloride ≥ 99.5%, methanol, pyridine ≥ 99.5%, potassium iodide ≥ 99.5%, iodine- ≥ 99.8%, and acetone were purchased from Carl Roth GmbH + Co. KG (Karlsruhe, Germany). Acetic acid 100% was purchased from AppliChem GmbH (Darmstadt, Germany); amyloglucodidase (from Aspergillus niger, 2000 U/mg) was purchased from Megazyme Ltd. (Wicklow, Ireland); hydrochloric acid 37% was purchased from Honeywell (Morristown, NJ, USA). Rye Samples.

Overall, 154 different samples from three different harvest years (2011–2013) were the basis for this approach. From harvest year 2012, 31 rye samples cultivated in Germany were taken into account for the present study. Of these 31 samples, 21 samples came from organic cultivation, eight samples from integrated cultivation, and eight samples were type flours. Out of these 31 samples, 13 samples were population rye (P), and 18 samples were hybrid rye (H). Eight different cultivars were present in this sample set. The six exemplarily selected rye samples presented in the figures were No. 1. cv. Baro (P); No. 2. cv. Brasetto (H); No. 3. cv. Minello (H); Nos. 4–6. cv. Palazzo (H).

All 31 rye samples were gently dried by simple ventilation in order to reach a final moisture content of ≤14%. A hammer mill (Laboratory Mill 3100) and a sieve insert (< 0.8 mm; Perten Instruments GmbH, Hamburg, Germany) were used for yielding wholemeal flours from the cleaned rye grains.

### 2.2. Methods

#### 2.2.1. Determination of Falling Number

Falling number (FN) was determined according to the Hagberg-Perten principle and the method collection of the *International Association for Cereal Science and Technology* (ICC method No. 107) [29]. This method is based on the characterization of the consistency of a meal or flour suspension in a boiling water bath and tested with a defined, specially prepared test body. FN corresponds to the time (in seconds) required by the falling body to completely penetrate the suspension. FN was determined using a Falling Number Apparatus 1800 (Perten Instruments GmbH, Hamburg, Germany); 25 mL of water was added per 7 g rye flour at a base moisture of 14%.

#### 2.2.2. Determination of Amylographic Data

The gelatinization properties of starch during heating were determined using an amylograph (Amylograph-E, Brabender GmbH & Co. KG, Duisburg, Germany) according to ICC method No. 126/1 [30]: A flour (80 g) or wholemeal (90 g) water (450 mL) suspension was prepared and heated at a constant heating rate (1.5 °C/min) in a measuring pot rotated at constant speed. The consistency of the resulting gel was continuously measured as torque and recorded in relative, device-related units (amylograph units, AE). The characteristic values of viscosity (in AE) and temperature (°C) in the gelatinization maximum were used to assess the gelatinization properties.

#### 2.2.3. Evaluation of Baking Quality

A modified standard baking test was applied, which allows the interpretation of the rheological data using the baking result [28]. In this baking experiment, wholemeal was used and the sourdough was replaced by a commercially available dough acidifier on a citric acid basis.

The standard baking test for rye flour was used to assess the baking and sensory properties of rye type flour. Processing was carried out as a sourdough baking test based on the Berlin short sour test (variant III of the standard baking test) [28]. The baking test is based on 800 g of wholemeal in relation to basic moisture of 14%. Water was added in relation to the measured water absorption of the respective wholemeal plus 12%. The baking test was performed with 1% dough acidifier (Ulmer Boerol, CSM Deutschland GmbH, Bingen am Rhein, Germany), 1.5% sodium chloride, and 1% pressed yeast, all calculated on wholemeal quantity.

The technical execution of the baking test is based on mechanical kneading with a small planetary mixer and a flat dough stirrer, manual preparation, and baking in a deck oven (Infra AE 506/26, Wachtel GmbH, Hilden, Germany). The use of a constant quantity of flour (14% moisture) requires a loss-free preparation of the dough, finally enabling the calculation of the volume yield related to flour. Baking in a box, as opposed to a released bread, increases the sensitivity of the statements with regard to loosening and poring. In addition to the basic evaluation, further tests were carried out according to the sensory characteristic groups (Table 1). The characteristics of the crumb, loosening, poring, haptic impressions, and chewing behavior were of a primary nature in the assessment.

For dough preparation, a planetary flat dough stirrer (Hobart N50) was used. The kneading time amounted to 10 min at 30 °C. The dough resting time was 45 min. For dough processing, two pieces per 50% of dough were matured at 32 °C (78% rel. humidity). Fermentation time amounted to 45 ± 5 min. The dough was baked at 210 °C for 55 min with two steam applications, the first for 1 s and the second for 3 s (vapor separation after 1 min).

The evaluation based on the physical data of the baked goods plus texture profile analysis was performed with an in-house trained sensory panel. The inclusion of aged bread (120 h) allowed the consideration of subsequently occurring crumb cracks and defects in the tactile and chewing impressions.

The maximum possible number of points for structure-relevant quality characteristics was 19 for breads using a dough acidifier and 20 for breads produced with sourdough. In this study, all breads were produced with a dough acidifier. A score of 16 points and above was considered good crumb quality, 12–15 points was considered medium quality, and 12 points or lower was considered poor baking quality. If crumb cracks were visible after 120 h, a devaluation by four points was made. Increased strength and reduced elasticity led to lower devaluations. According to a previously developed score system [31], specific scores were calculated on the basis of the score 24 and 120 h after baking divided by the maximum possible score. Information on this scoring is given in Table S1.

#### 2.2.4. Water Absorption of Rye-Type Flours

In order to determine the water absorption of rye-type flours in the standard baking test, the equivalent of 300 g flour with 14% moisture was mixed in the farinograph while the kneader was running with the amount of water absorption to be expected. Within 10 min., the final viscosity of the kneading curve must be 300 ± 10 FU (farinogram units). The amount of water added must be adjusted within the first 5 min. When necessary, kneading has to be done again and again until the correct water addition is determined [30]. For this study, a method was developed for determining water absorption for the baking test for rye wholemeal by the Mixolab system. The method was verified by comparing the water absorption calculated with the water absorption determined by the farinograph.

#### 2.2.5. Separation of the Ingredients of Rye Milling Products

Rye flours were subsequently fractionated/separated regarding their density in order to analyze the pure starch granules or, more specifically, proteins and hemicelluloses adhering to the granules. Consequently, the resulting change in starch gelatinization behavior in different quality classes can be studied. Furthermore, this technique allows us to analyze and quantify insoluble components, e.g., proteins and hemicelluloses, separatedly from starch. It can be assumed that these substances have, in particular, a great influence on the water balance in rye dough and on the transition to crumb. Therefore, it is necessary to study changes in the compositions of these components in different quality classes.

**Figure 1:**
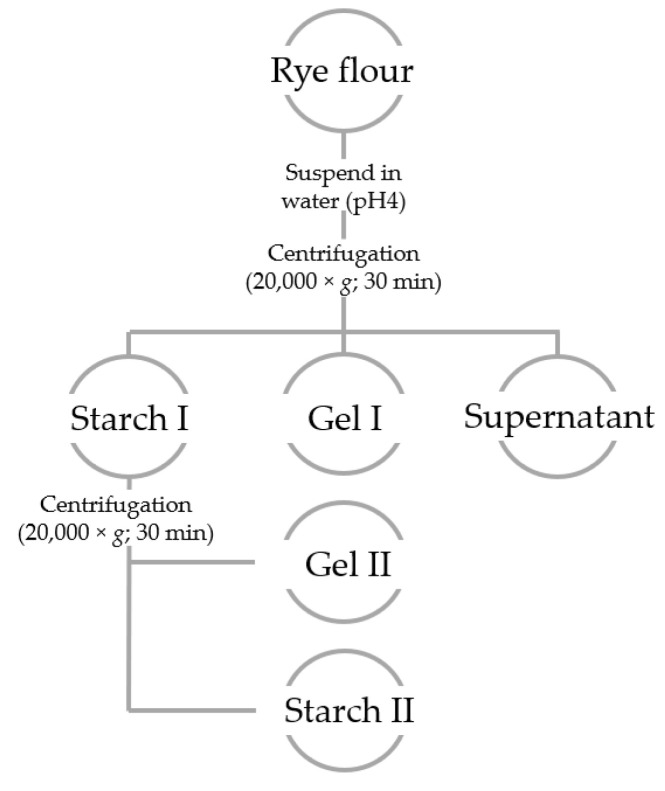
Separation scheme of the ingredients of rye milling products by centrifugation.

As rye-based doughs usually have a pH value <5, the separation of the ingredients was also carried out at a lower pH value. The sample processing procedure is shown schematically in Figure 1.

The grinding products were suspended in a mass ratio of 1:10 with demineralized water or water adjusted to pH 4. Swelling and dissolving processes took place within the first 2 h at room temperature. Afterward, a separation with regard to density was carried out by centrifugation (20,000× *g*; 30 min), leading to the three follow-up phases Gel I, Gel II, and Starch II (Figure 1).

The heaviest phase contained the large fractions of starch grains (Starch I). The middle phase was Gel I, which included limited swellable proteins and hemicelluloses and the fraction of small starch granules. This fraction of starch could be largely separated by repeated washing and centrifugation (leading to phases Starch II and Gel II). Starch II contained the smaller starch grains, and Gel II was a starch-free phase, mainly consisting of hemicelluloses and proteins. The supernatant(s) contained all soluble ingredients, especially soluble proteins and dietary fibers.

#### 2.2.6. Analysis of the Main Compounds

For the analysis of the main components, dry matter was determined at 130 °C according to a method described in the method collection of the *German Food and Feed Code* (Method No. L1601–1, § 64 *Lebensmittel-und Futtermittelgesetzbuch*—LFGB), and crude protein was determined by the Kjeldahl method according to Method No. L06-00-7 (§ 64 LFGB) [32]. Starch content was determined polarimetrically and enzymatically according to the specifications of the manufacturer (R-Biopharm AG, Darmstadt, Germany), and the sugar content was analyzed by high performance liquid chromatography (HPLC) equipped with a refractometric detector (limit of detection sucrose: 0.2 g/100g) according to ISO/IEC 17025:2005 [33].

#### 2.2.7. Determination of Glucan/Pentosan Content (Soluble Fraction)

For the determination of the soluble fraction of glucans and pentosans, a modified method according to § 64 LFGB (Method No. L 00.00-13) was applied.

For the calibration curves, commercially available β-glucan (200.0 mg) was dissolved in boiling water (80 mL). After cooling to room temperature, the solution was filled up to 100 mL. A dilution series (2 to 0.1 mg L^−1^) was prepared using 1, 0.5, 0.25, 0.1, or 0.05 mL of the solution. For the monomers of the pentosans, arabinose and xylose (50.0 mg each) were dissolved in water (50 mL) and consecutive dilution series were prepared.

For fat separation, the sample (2.00 g) was dissolved in water (23 mL). To remove grease, a mixture of acetone and dichloromethane (9/1, *v*/*v*; 40 mL) was added and heated to 40 °C for 10 min. Centrifugation (1878× *g*) yielded a residue that was extracted a second time. The remaining residue was washed twice with ethanol (10 mL, 70%) and centrifuged (1878× *g*) repeatedly. The remaining residue was dissolved in water (90 mL; 30 min, 90 °C) and filled up to 100 mL after cooling to room temperature.

To degrade the starch enzymatically, 30 mL of the fat free solution was mixed with sodium chloride solution (0.1 mL, saturated), α-amylase solution (1.5 mL; 500 KU mL^−1^; EC 3.2.1.1), and amyloglucosidase solution (0.5 mL; 200 U mL^−1^; EC 3.2.1.3) and kept in a water bath at 60 °C for 90 min.

After starch degradation, the solution was mixed with saturated sodium chloride solution (2 mL) and sulfosalicylic acid (4 mL, 25%, *w*/*w*) for protein precipitation. After 10 min., the precipitated components were separated by centrifugation (1878× *g*) and the supernatant was used for analysis.

The target polysaccharides (β-glucan, pentosans) were precipitated with ethanol (75 mL, absolute). After 14 h, the gelatinous precipitate was separated by centrifugation (1878× *g*), washed three times with ethanol and once with methanol, and dried for 30 min at 105 °C.

The residue was dissolved in methanolic hydrochloric acid (1 mL, 2 mol L^−1^) and incubated in a carefully closed screw vessel at 100 °C for 4 h. After cooling, a solution of i-erythritol (0.33 mg) and sorbitol (0.500 mg) was added as internal standard (dissolved in 1 mL methanol), as well as pyridine (0.05 mL). After removing the solvent in a vacuum, the oily residue was used for derivatization.

For the derivatization, the residue was dissolved in pyridine (0.5 mL) mixed with N,O-bis-(trimethylsilyl)-acetamide (BSA, 0.25 mL), trimethylchlorosilane (0.05 mL), and 1,1,2-trichlorotrifluoroethane (0.25 mL) and incubated in a carefully closed screw-cap vessel for 1 h at 60 °C. In case of turbidity, further BSA was added until it was dissolved. After membrane filtration and, when necessary, dilution with 1,1,2-trichlorotrifluoroethane, this solution was used for measurements by gaschromatography coupled with a flame-ionization detector (GC-FID).

The GC-FID measurements were performed on a 5890 Series II instrument (Hewlett Packard Corp., Palo Alto, CA, USA) using a DB-5 column (30 m, i.d. 0.32 mm), split 1:15, with nitrogen as the carrier gas. The following conditions were applied: temperature program from 120 to 220 °C at 4 °C min^−1^ and an injection volume of 1 µL.

For evaluation, the sugar-specific area correction factor F_k_ was calculated and the total amount of sugar was quantified.

#### 2.2.8. Determination of Pentosan Content

For determining the total content of pentosans, the phloroglucinol method, according to Douglas (1981), followed by an optimized version, according to Kiszonas et al. (2012), was applied [34].

At least 10 mg of the sample was weighed into heat-resistant sealable glass reaction tubes. The weight of the sample was chosen so that the difference in absorption measured after the reaction was at least 0.1 and not more than 0.7. The absorbance at 558 nm must not exceed 1.0. To each sample, 2 mL of distilled water was added.

Calibration standards (0, 0.025, 0.05, 0.075, 0.1, 0.125, 0.15 g/L) were also prepared in sealable reaction tubes. The reagent solution was prepared by dissolving 1 g phloroglucinol in 10 mL ethanol and subsequently adding 1.515 mL water, 167 mL of acetic acid, and 4.545 mL concentrated hydrochloric acid (37%).

Every 15 s, 10 mL of the reagent solution was pipetted to the samples or 5 mL of the reagent solution was pipetted to each calibration standard. All preparations were then well vortexed at equal intervals (15 s) in the same order and incubated in a boiling water bath for 25 min. After the reaction, all samples were removed from the water bath and cooled in an ice bath for 10 min. The absorbance at the wavelengths 558 and 505 nm was determined by means of a photometer (Specord 40, Analytik Jena AG, Jena, Germany) at the corresponding 15 s intervals. The absorption difference of these two values was used for quantification. The content of xylose or pentosan in the sample was determined using the calibration curve (Figure S1).

#### 2.2.9. Determination of the Starch—Amylose-to-Amylopectin Ratio

A commercial enzyme kit (K-AMYL 07/1, Megazyme Ltd., Wicklow, Ireland) was used to determine the ratio of amylose to amylopectin in starch. Procedures were done in accordance with the manufacturer’s protocol.

#### 2.2.10. Determination of Conversion Enthalpy

Enthalpy at gelatinization of starch or denaturation of proteins is a measure of how much energy is required to cause the phase transition. The more compact the starch grains, the higher the enthalpy of gelatinization. The higher the proportion of undenatured proteins, the higher the denaturation enthalpy. The conversion of enthalpy was determined by thermal analysis (MDSC^®^ 2920, TA Instruments Inc., New Castle, DE, USA). For this purpose, starch samples (Starch I and Starch II) were weighed into a pan, and an excess of water (three to four times the initial starch mass) was added. The crucible was hermetically sealed.

The heat flow required to cause the gelatinization reaction was measured, and the enthalpy of gelatinization was calculated using the weight transformations.

#### 2.2.11. Partial Gelatinization

Generally, processing does not take place with an excess of water but under the conditions of a limited amount of available water. Here, the accessibility of starch to water and the competition for water absorption, especially by hemicelluloses, influence the gelatinization behavior.

For this reason, partial gelatinization of the starch with 45% water was carried out. To avoid water loss through evaporation and evaporation during the gelatinization process, starch and water were mixed and hermetically sealed in a vial (10 mL) and incubated for at least 2 h. Heating was carried out in a water bath at 95 °C for 30 min. After cooling to room temperature, the vials were opened, and the partially gelatinized starch was homogenized with a mortar. Analytical tests on partially gelatinized starch were carried out not earlier than 3 h and not later than 24 h after the heat treatment.

#### 2.2.12. Light Microscopic Evaluation of Gelatinization

The state of gelatinization can be visualized microscopically after staining with iodine–potassium iodide. For the analysis, a Leitz Aristoplan light microscope (Leica Microsystems GmbH, Wetzlar, Germany) was used. The lower the degree of gelatinization of the starch, the more intensive the dark coloring. Well-gelatinized starch appears light-brownish and transparent.

#### 2.2.13. Confocal Laser Scanning Microscopy

Confocal laser scanning microscopy (CLSM) is able to distinguish groups of substances (proteins, lipids, carbohydrates) in a matrix. Laser light is focused on a small volume element in the sample, and a confocal point detector was used to record the resulting signal. In this way, only an image of the focused plane was generated. The lens functioned as both a condenser and a collector. To be able to examine a sufficiently large area of a sample, the examination plane was scanned. In order to make the substances in the observation plane visible, it was necessary that they were doped with a fluorescent substance that can be excited by laser light. Light of a complementary wavelength was then emitted. To illustrate the coating of the starch surface, the proteins were doped with Rhodamine R (absorption 570 nm; emission 590 nm). Protein structures appeared red. CLSM images were generated with an Eclipse E600 microscope (Nikon Imaging Japan Inc., Tokyo, Japan).

### 2.3. Data Analysis

All analytical measurements were carried out in duplicates. The presented data show the mean values. In addition, measurement uncertainties of the individual methods are indicated as error bars. In the last ten years, rye harvest has been evaluated using several hundred rye samples and a (modified) standard baking test [28]. However, this test is very laborious, time-consuming, and not applicable to the milling and bakery industries. The mean values given in the figures are, therefore, not representative of biological (and baking) variability. Due to the large number of samples and the usual methodological procedures and analytical strategies, a double determination of the parameters was done. Error bars give an impression of the analytical variability.

The differentiation of rye bread in different quality classes is based on the point loss of the sensory properties of the bread according to a (modified) standard baking test, as described in Section 2.2.3 [31]. The maximum number of points achievable is 20 points. Points were assigned for volume/shape (max. two points), crust (max. two points), crumb (max. eleven points), smell/taste (max. four points), and acidity level (max. one point). The classification into quality classes was made according to the following scheme: very good (20–19 points), good (18–16 points), medium (15–12 points), and poor (<12 points). Sensory characteristics were measured after 24 and 120 h.

## 3. Results and Discussion

### 3.1. Sample Selection

To investigate the coherence between rye flour ingredients and the quality of baked goods, the main components (starch, proteins, pentosans, and glucans) of selected rye flours were quantified. In order to draw conclusions from the results of these quantifications on bread quality, a standardized rye flour baking test was performed to categorize the different rye flour samples into the quality classes of good, medium, and poor quality rye bread [31]. Consequently, differences in the quantification results within the different quality classes can help us to identify the parameters responsible for poor quality rye bread.

To assign which rye flour sample should be subjected to more precise characterization, only the baking volume and structure-relevant sensory properties (looseness/pore pattern, elasticity, and texture) were included. These characteristics were determined 24 and 120 h after baking. Three quality classes were formed (in the following part, red—“poor”, yellow—“ medium”, and green—“good”) according to the cumulative overall result. As the aim is to find flour characteristics that influence baking quality independently of FN and amylographic data, the samples of the quality classes were selected in clearly different ranges of FN. Out of 31 flour samples, two were assigned to good baking quality, two to poor baking quality, and 27 were assigned to medium baking quality. From the 27 samples with a medium baking quality, two representatives that differed significantly in their cereal analytical characteristics (e.g., FN and amylographic data) were selected. Six samples, selected as exemplary representatives, are described in Table 1.

The results presented in Table 1 show a high gelatinization maximum for samples of “poor” quality (Samples 5 and 6) and one “medium” quality sample (Sample 4). These samples all belong to the cultivar Palazzo. Additionally, a sample with a good baking quality (Sample 2), belonging to the cultivar Brasetto, also showed a high gelatinization maximum, within the same range as the samples from cv. Brasetto. The gelatinization temperature differed from 66.9 to 74.0 °C between the different samples regardless of variety or baking quality.

### 3.2. Quantification of Main Components

Initially, the main compounds were quantified directly from rye wholemeal. The results showed that protein and total starch content (Figures S2 and S3) were largely independent of baking quality but were inversely proportional to each other within the quality classes of “good” and “medium”. This means that samples with lower starch content had higher protein content and vice versa. However, the differences in starch content between the different samples were up to 3.8 g/100 g dry matter (dm) from the lowest to the highest starch content. This tendency was to be expected because of the opposed dependency between nitrogen and carbon metabolism via the tricarboxylic acid (TCA) cycle, more specifically 2-oxoglutarate, which functions either as carbon skeleton for nitrogen assimilation or is degraded by the 2-oxoglutarate dehydrogenase complex in the TCA cycle [35].

**Figure 2:**
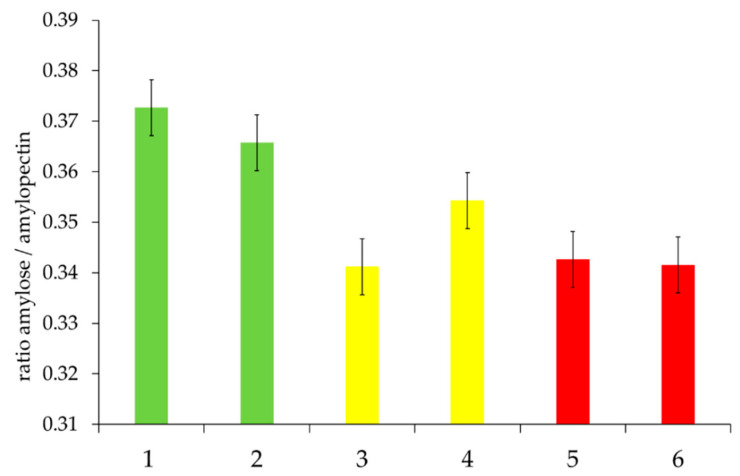
Ratio of amylose and amylopectin in the selected rye flour samples, depending on “good” to “medium” to “poor” baking quality and the corresponding falling number (FN) within the individual baking quality classes (here: first samples were always assigned the lower FN and second samples the higher FN). Sample description: see Table 1. Bars represent the mean value of two replicates; error bars represent the confidence interval.

However, the total starch content should not be the only starch parameter analyzed as starch is a polysaccharide consisting of amylose and amylopectin units. These two components, composed of glucose units, differ in their linkage and, thus, in their physicochemical properties as well. The role of amylose and amylopectin with regard to the staling of bread has already been studied, revealing a loss of crystallinity for amylopectin during baking. In fresh bread, amylopectin is in an amorphous state. During the aging process, the bread becomes more rigid due to the reorganization of the crystalline amylopectin structure [36]. The properties of amylose and amylopectin only become effective after the destruction of the granules’ structure due to swelling and gelatinization (and partial hydrolysis). Consequently, the selected rye flours were also tested with regard to their ratio of amylose and amylopectin (Figure 2).

The characterized classes of baking quality showed a tendency to have higher amounts of amylose for “good” quality rye flours, whereas “medium” and “poor” quality flours provided higher amounts of amylopectin. During the gelatinization process, amorphous regions absorb water, destabilizing the crystallites, leading to increased swelling of the granules and the loss of the crystalline structure by melting. The swelling power of starch granules is mainly attributed to amylopectin [37]. Meanwhile, amylose diffuses out of the granules and builds up a continuous network [38]. Thus, a decreased content of amylose could upset the balance of this process. Amylose also has higher water solubility in comparison to amylopectin. The lower amount of amylose might result in decreased water transport through the different starch regions and, thus, in inadequate gelatinization.

**Figure 3:**
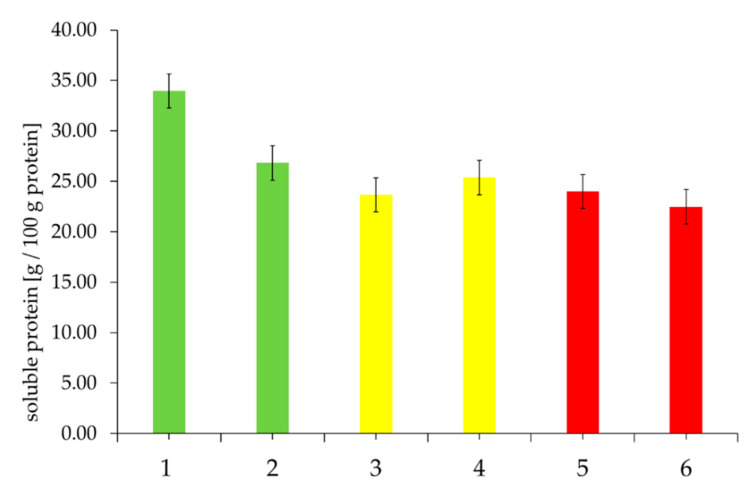
Proportion of soluble proteins (%) in relation to total protein content of the selected rye flour samples, depending on “good” to “medium” to “poor” baking quality and the corresponding falling number (FN) within the individual baking quality classes (here: first samples were always assigned the FN and second samples the higher FN). Sample description: see Table 1. Bars represent the mean value of two replicates; error bars represent the confidence interval.

However, the percentage of soluble proteins in the selected rye flours, as shown in Figure 3, indicates the tendency that good quality rye flour (according to the baking test) has more soluble proteins in relation to total protein content.

**Figure 4:**
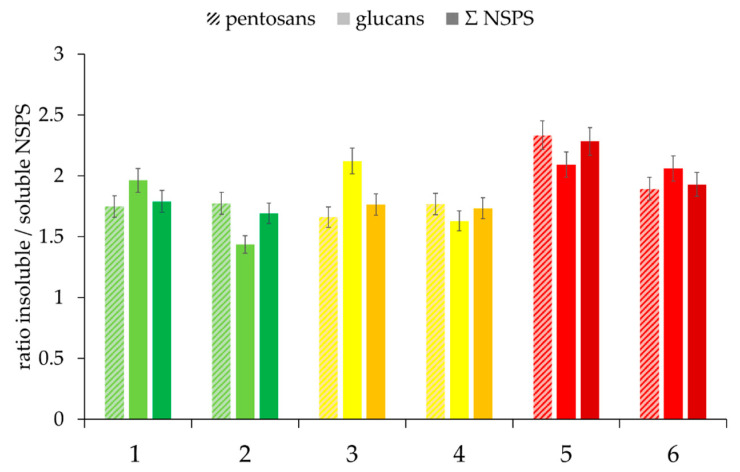
Ratio of insoluble-to-soluble NSPS of the selected rye flour samples, depending on “good” to “medium” to “poor” baking quality and the corresponding falling number (FN) within the individual classes (here: first samples were always assigned the lower FN and second samples the higher FN). Sample description: see Table 1. Bars represent the mean value of two replicates; error bars represent the confidence interval.

The contents of soluble and insoluble pentosans and glucans, as well as the sum of NSPS, measured directly from rye flour, showed no correlation with baking quality. However, the ratio of insoluble-to-soluble NSPS was significantly higher for the poor baking quality class (Figure 4). However, the samples preclassified as good and medium quality could not be further differentiated with regard to this parameter.

With regard to cultivars of the rye samples, Samples 5 and 6 (both cv. Palazzo) with poor baking quality and the cv. Palazzo sample with medium baking quality seem to have similar ratios of amylose/amylopectin, as well as soluble protein content, but differ in the amount of NSPS. Sample 5 displays approx. 14% higher content of NSPS in comparison to Sample 6 and an approx. 24% higher ratio in comparison to Sample 5. Although, at first, it seems likely that baking quality could depend on cultivar, these observations suggest opposite and more complex dependencies. This is also obvious from the following analyses of the chemical composition of those six exemplarily selected rye samples.

As mentioned before, this topic has an intense history. In the last ten years, rye harvests have been evaluated using several hundred rye samples and a (modified) standard baking test [28]. As this test is very laborious, time-consuming, and not applicable to the milling and bakery industries, our ideas are aimed at identifying simpler, easy-to-analyze parameters for rye quality evaluation and a certain predictability of baking results. Consequently, this study aims at a broader characterization of selected quality-defining parameters using a selection of samples differing in baking quality. However, the quantification of the main components directly from rye wholemeal did not allow us to draw any conclusions on baking quality so far. This is the main drawback of this topic, which has encouraged researchers to find methods, quality criteria, or even biomarkers for predicting the appropriateness of rye samples for use in the baking industry. Consequently, the flours were separated into individual components. Soluble, limited swellable, and insoluble components of rye flour most probably contribute in different ways to the functional properties of dough and bread. Therefore, rye flours were subsequently fractionated by density according to the centrifugation scheme in Figure 1.

The following fractions/phases were obtained:

- Supernatant (water-soluble components)
- Starch I (fraction of larger starch granules or granules of higher density)
- Starch II (fraction of smaller starch granules or granules of lower density)
- Gel (insoluble proteins and hemicelluloses)

By separating the samples into different phases, their individual contribution, as well as their limitations regarding the formation of dough and the production of a good quality rye bread, could be characterized.

### 3.3. Characterization of the Starch Phases of Selected Rye Flours

Starch is the main component of rye flour; it has a decisive influence on baking quality as starch gelatinization is the most important outcome during dough preparation. Starch granules consist of amorphous and crystalline regions of amylose and amylopectin. They are surrounded by a protein layer, through which all interactions of native starch granules with each other or with other compounds (e.g., proteins or hemicelluloses) take place. Overall, prerequisites for starch gelatinization are the availability of water and the accessibility of starch granules. However, as already described above, proteins in rye do not form a gluten-like network that allows an adequate formation of pores. In rye bread, NSPS, mainly hemicelluloses, contribute a large part to baking properties. Insoluble pentosans contribute to water retention in the dough, whereas soluble pentosans have a positive effect on dough loosening, crumb, and bread volume. It becomes obvious that starch gelatinization is largely dependent on the proteins and hemicelluloses surrounding the granules, as well as the granule structure itself.

**Figure 5:**
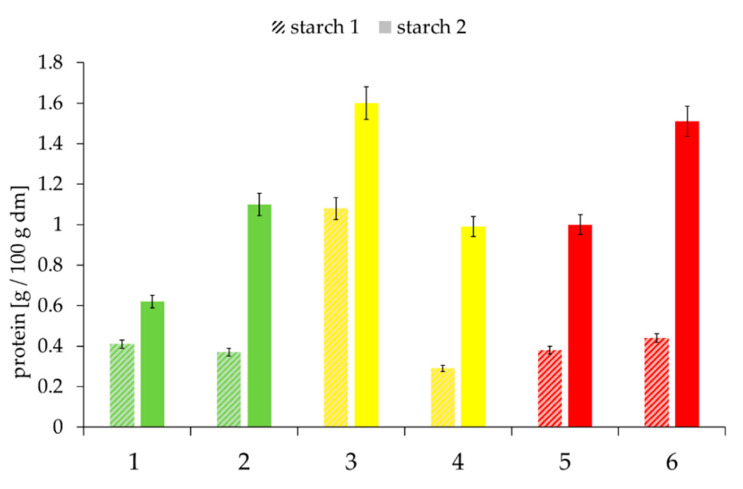
Protein content of the starch phases of the selected samples, depending on “good” to “medium” to “poor” baking quality and the falling number (FN) within the individual baking quality classes (here: first samples were always assigned the lower FN and second samples the higher FN). Sample description: see Table 1. Bars represent the mean value of two replicates; error bars represent the confidence interval.

**Figure 6:**
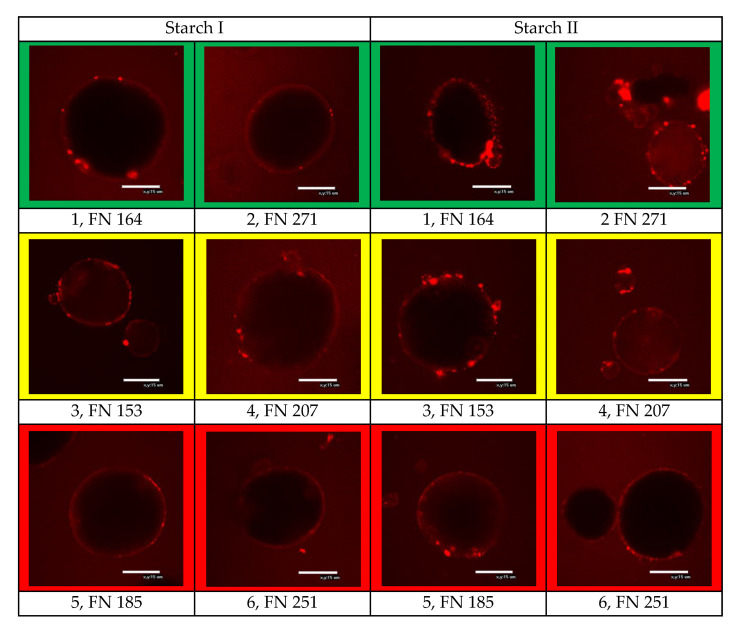
Microscopic images of protein distribution and coating (red spots and aura) of the starch granules (black balls) surfaces of the selected samples, depending on “good” to “medium” to “poor” baking quality and the corresponding falling number (FN) within the individual baking quality classes (here: first samples were always assigned the lower FN and second samples the higher FN). Sample description: see Table 1; bar = 15 µm.

As already mentioned, the interactions between native starch granules and between starch granules and other components are restricted by the protein layer on the granules’ surface. For gelatinization and other interactions with other components, whether the starch granules are completely coated with proteins, thereby sealing the granules, is crucial. Therefore, the amount of proteins adhering to the surface of the starch was determined quantitatively (Figure 5) and characterized microscopically (Figure 6).

In general, there was a large variance in the protein content of the two starch phases. As phase Starch II was obtained from the gel phase by repeated washing steps, it can be concluded that the larger protein deposits (Figure 6) were also derived from more intensive interactions with insoluble proteins and hemicelluloses of the gel phase.

Proteins were not homogeneously distributed on the starch grain surface, as the CLSM images (Figure 6; proteins in red color) illustrate. High protein contents are mainly caused by dense agglomerates. However, these are only of limited importance to the accessibility of starch to water if the surfaces are not completely dense.

**Figure 7:**
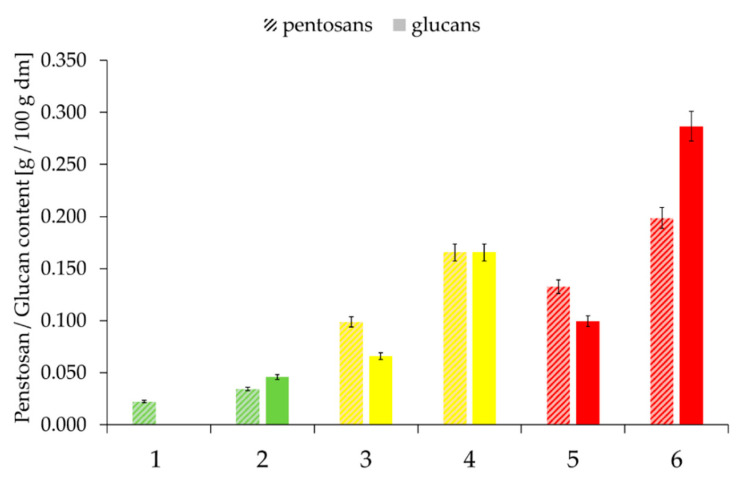
Pentosan and glucan contents in the Starch II phase of the selected samples, depending on “good” to “medium” to “poor” baking quality and the corresponding falling number (FN) within the individual baking quality classes (here: first samples were always assigned the lower FN and second samples the higher FN). Sample description: see Table 1. Bars represent the mean value of two replicates; error bars represent the confidence interval.

Previous studies have shown that proteins can form complexes with hemicelluloses such as arabinoxylan [39,40]. Consequently, pentosans and glucans were also analyzed in the individual starch phases (Figure 7) to determine whether the agglomerates on the starch granules’ surface might also contain insoluble hemicelluloses. A higher content of insoluble NSPSs, especially hemicelluloses, could negatively influence starch gelatinization due to the competition for free water.

In the Starch I phase, pentosans were not detectable in any of the samples analyzed (limit of determination 0.01 g/100 g). The concentration of glucans was also rather low (0.01–0.03 g/g dm). This is in accordance with the CLSM images (Figure 6), which show protein agglomerates in a low abundance for the Starch I phase.

The Starch II phase contained considerably more NSPSs (Figure 7), allowing us to draw a conclusion on good quality flour in comparison to medium and poor quality flour. The content of NSPSs in Starch II amounted to less than 0.05 g/100 g. The total amount of NSPSs in Starch II is also related to FN. Within the two samples of the three quality classes, the amount of NSPSs was higher within the sample with a higher FN.

So far, the results suggeste that an insufficient starch gelatinization leads to an unsatisfying bread quality, probably caused by interactions between proteins and hemicelluloses on the surfaces of the starch grains. To further understand and evaluate the gelatinization process of rye flours resulting in different quality baked goods, gelatinization enthalpy was measured by differential scanning calorimetry. No differences between the classes of baking quality or dependencies on FN could be identified. This means that the native starch is easily accessible to water in the case of a surplus of water. However, this result does not necessarily apply to gelatinization with a limited water supply. Here, denaturation of the proteins on the starch grain’s surface in interaction with the hemicelluloses may well result in a reduction in the accessibility of starch grains, which could not be detected in the case of excess water.

**Figure 8:**
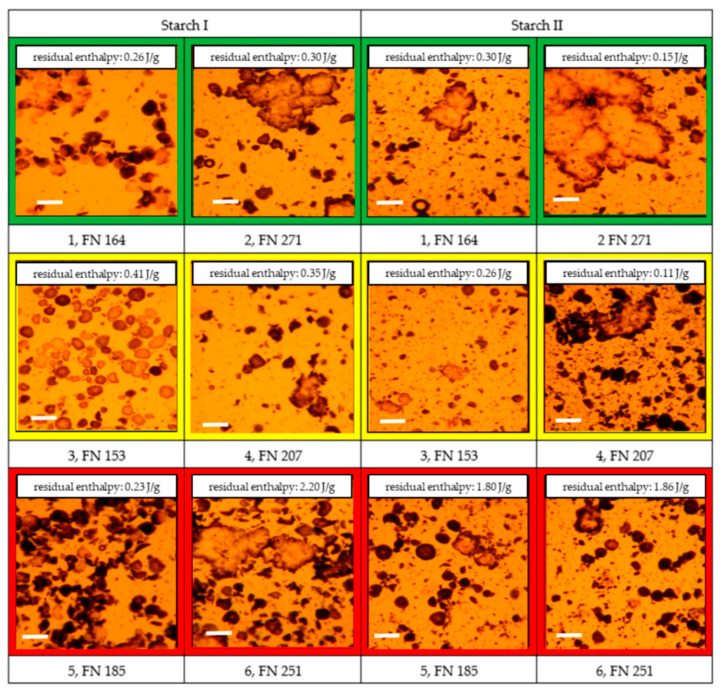
Light microscopic images of the state of starch phases after partial gelatinization of the selected samples, depending on “good” to “medium” to “poor” baking quality and the corresponding falling number (FN) within the individual baking quality classes (here: first samples were always assigned the lower FN and second samples the higher FN). Sample description: see Table 1. Bar = 100 µm.

Therefore, the starch phases were subjected to partial gelatinization with a water content of 45%, which approximately corresponds to real conditions in rye dough. The degree of starch gelatinization and the ability to form structure-stabilizing agglomerates were qualitatively illustrated using light microscopy by staining with iodine–potassium iodide (Figure 8).

Well gelatinized starch is characterized by low coloration. The darker the color, the more compact the starch granules after partial gelatinization. The light microscopy images of the good and medium quality rye flours indicated more compact starch granules for the higher FN. The images for poor quality rye flour showed a dense distribution of compact starch granules after partial gelatinization, especially in the Starch I phase, illustrating poor gelatinization.

**Figure 9:**
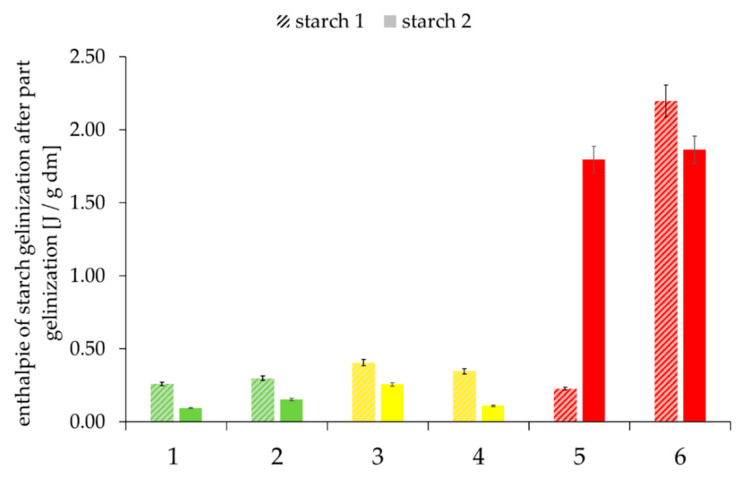
Residual enthalpy of partially gelatinized starch with excess water of the selected samples, depending on “good” to “medium” to “poor” baking quality and the corresponding falling number (FN) within the individual baking quality classes (here: first samples were always assigned the lower FN and second samples the higher FN). Sample description: see Table 1. Bars represent the mean value of two replicates; error bars represent the confidence interval.

Whether a lack of water or a thermal modification of the surface properties of starch granules inhibits gelatinization can be analyzed by determining the enthalpy of gelatinization in the case of excess water (Figure 9).

Low enthalpies occur either when starch is already well gelatinized or has no access to water. Even in a partially gelatinized state, high enthalpies occur when a lack of water (caused by the competition with other components such as hemicelluloses) hinders starch gelatinization, although the starch is, in principle, accessible to water.

There were two obvious examples (Starch II–Sample 4 and Starch I–Sample 5) in which the optically visible gelatinization state did not correspond to the measured residual enthalpy. However, further analysis results were only conditionally suitable to explain this phenomenon. It can therefore be assumed that quality-relevant modifications of starch granule surfaces also occur during partial gelatinization under the condition of reduced water supply.

### 3.4. Composition of the Gel Phase of the Selected Rye Flours

The components of the gel phases probably have a much greater significance within the processes of structural change in the production of rye products than previously assumed. The essential components of the gel phase are insoluble proteins and hemicelluloses, which have high molecular masses and a complex structure and form a soft viscoelastic network. Sárossy et al. (2011) found very similar molecular masses for water-extractable and nonextractable rye hemicelluloses of about 700 kDa. Their molecular weight can range from 200 to 900 kDa, depending on branching as well as the analytical method (in most cases, use of different size exclusion chromatography (SEC) systems), the eluent, and, especially, the method used for calculation [41]. The most abundant proteins in the gel phase are rye storage proteins, called secalins. Depending on single secalins, molecular weight can range from 32 to 90 kDa [42]. It can be assumed that it is precisely these components that have a major influence on the water balance in rye dough and the transition to crumb as hemicelluloses bind an immense amount of water and proteins are able to release water through heat denaturation. In addition, proteins and hemicelluloses can form complexes that influence functional properties. Previously published studies suggest that interactions between these components influence the technological properties of dough as well as bread texture [43,44]. A complex formation could, for example, lead to a modification of protein denaturation kinetics, which results in reduced release and, therefore, availability of water for the gelatinization process.

**Figure 10:**
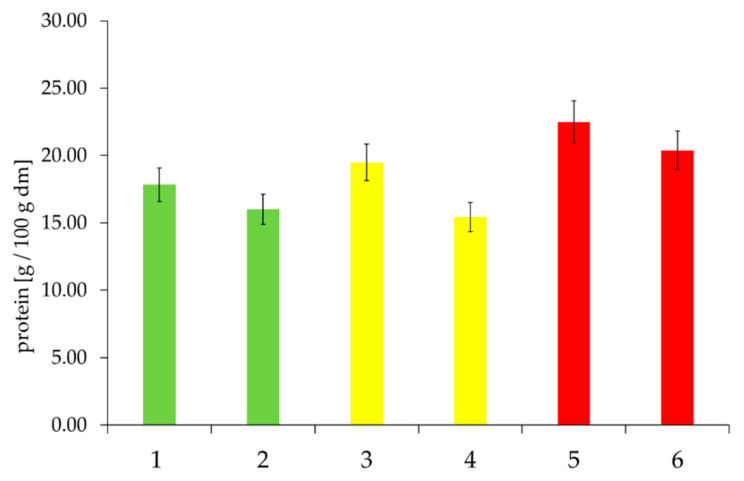
Protein content in the gel phases of the selected samples, depending on “good” to “medium” to “poor” baking quality and the corresponding falling number (FN) within the individual baking quality classes (here: first samples were always assigned the lower FN and second samples the higher FN). Sample description: see Table 1. Bars represent the mean value of two replicates; error bars represent the confidence interval.

**Figure 11:**
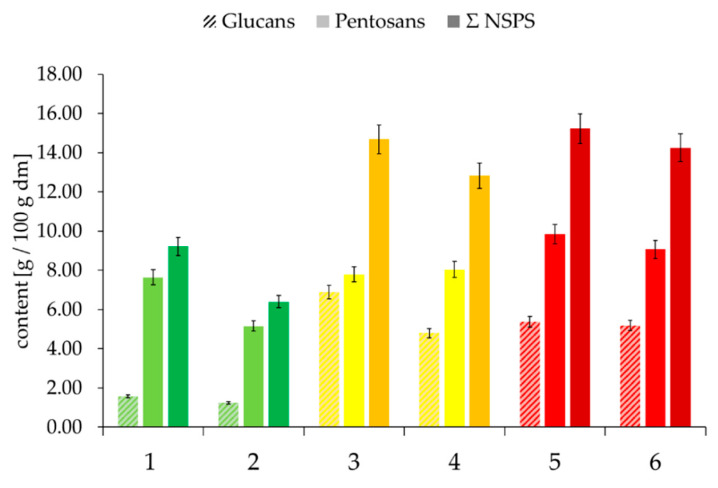
Content of NSPSs in the gel phases of the selected samples, depending on “good” to “medium” to “poor” baking quality and the corresponding falling number (FN) within the individual baking quality classes (here: first samples were always assigned the lower FN and second samples the higher FN). Sample description: see Table 1. Bars represent the mean value of two replicates; error bars represent the confidence interval.

The results of the quantification of proteins and NSPSs are illustrated in Figure 10 and Figure 11. The determination of protein and NSPS contents showed that differences between the quality classes could be identified. In the case of protein content, the influence of FN was also obvious within the quality classes (Figure 10). The “poor” quality class showed an increased proportion of proteins and pentosans compared with the other quality classes. Within the quality classes, the samples with a higher FN had lower protein content.

With regard to glucan content and total NSPS content (Figure 11), the “good” quality class differed significantly from the “medium” and “poor” quality classes. These results indicate that higher protein and NSPS contents in the gel phase are connected to poorer bread quality. As already mentioned above, these components have a significant influence on technological properties and bread structure. It was found that high concentrations of the hemicellulose arabinoxylan prevent the formation of a protein network in wheat, leading to decreased dough elasticity and a harder crumb. Rye does not form such a protein network. A reason for this might be because rye naturally has much higher arabinoxylan concentrations (5–7%) in comparison to wheat (1.5–2.5%) [45,46]. Beck et al. (2011) found that the addition of the most prominent protein crosslinking enzyme transglutaminase, up to a certain concentration, has a positive influence on rye bread volume and crumb elasticity [47]. Transglutaminase catalyzes crosslinking between protein-bound glutamine and lysine residues, as well as linkages of amines to glutamine and deamidation of glutamines, thereby enabling the formation of a protein network. Grossman et al. (2016) found that the addition of both transglutaminase and xylanase, with the latter being an arabinoxylan-degrading enzyme, improves bread quality even more, although the degradation of arabinoxylan did not seem to influence protein–protein interactions. Those studies postulate the synergistic effect of transglutaminase, resulting in increased rye bread volume. In this case, xylanase degrades arabinoxylan, producing a higher proportion of water-soluble arabinoxylans [43]. Buksa et al. (2016) found that arabinoxylan-protein-complexes in rye can be correlated with comparatively higher bread volume [48]. However, the present results suggest that differentiation between water-soluble and water-insoluble hemicelluloses must be made, as insoluble hemicelluloses seem to have a negative effect on bread quality.

**Figure 12:**
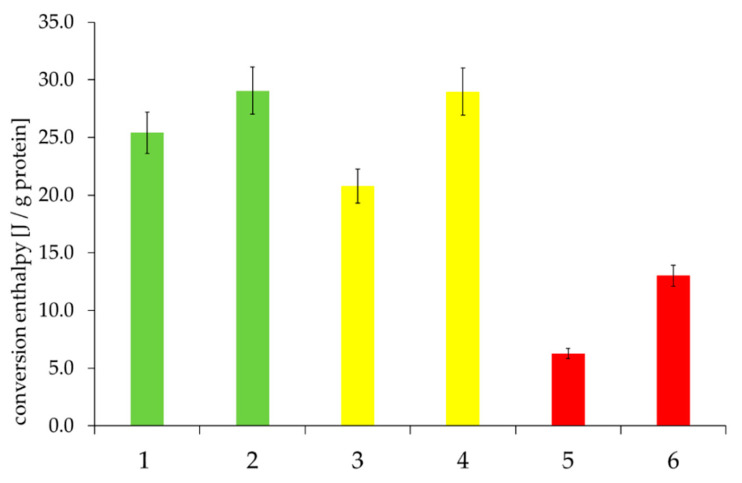
Conversion enthalpy in the gel phases of the selected samples, depending on “good” to “medium” to “poor” baking quality and the corresponding falling number (FN) within the individual baking quality classes (here: first samples were always assigned the lower FN and second samples the higher FN). Sample description: see Table 1. Bars represent the mean value of two replicates; error bars represent the confidence interval.

Although the gel phase of the “poor” quality class had significantly higher protein contents, the lowest conversion enthalpy (Figure 12) was found here. Conversion enthalpy in the gel phase describes the denaturation process of the proteins. The lower the enthalpy, the lower the amount of denatured proteins. This supports the hypothesis that the interactions between proteins and insoluble hemicelluloses (e.g., arabinoxylan) have a great influence on the functional properties of rye components, for example, the obstruction of protein denaturation (leading to a lack of free water for gelatinization) due to complex interactions with other components like hemicelluloses. The more intensive these interactions, the more influenced the baking quality, especially by interfering with the water balance.

Buksa et al. (2016) found possible evidence for the formation of high molecular weight complexes consisting of proteins and arabinoxylans, the most abundant pentosan in rye [21]. These studies suggest that the covalent linkages are formed with ferulic acid as the crosslinker, which is often bound to arabinose residues of arabinoxylans. Interactions between proteins and phenolic compounds such as ferulic acid have already been extensively studied, revealing possible covalent bonds between ferulic acid (derivatives) and amino acids such as lysine, cysteine, methionine, and tryptophan [49]. Piber and Koehler (2005) also identified dehydroferulic acid-tyrosine as a possible compound in heterocrosslinking between proteins and carbohydrates [50]. Gellrich et al. (2003) showed that the compositions of the protein fractions (according to Osborne solubility) differed in individual rye-type flours. Moreover, they found that the quantity of different secalin types, which are abundantly present in the gel phase, can vary in different flour types [23]. It is possible that the different protein types have different affinities for complex formation with ferulic acid and hemicelluloses due to their amino acid sequence and corresponding spatial structure.

An overview of the results from the quantitative analysis is shown in Table S2.

## 4. Conclusions

In order to analyze the relationship between the composition of the main components and the resulting quality of rye breads, 31 rye flour samples from a 2012 harvest were categorized into three different quality classes (good, medium, and poor) according to a traditional standard baking test. From a selection of prominent samples of these quality classes, starch (fractions), proteins, and NSPSs were quantified.

The quantitative measurements directly from the rye flours did not lead to a clear prediction of the quality of baked goods. Although the samples with poor baking quality belonged to the same cultivar, the analysis of the chemical compositions showed that cultivars do not necessarily correlate with chemical composition as they are significantly influenced by ecophysiological conditions. However, rye flours of poor baking quality showed a higher ratio of insoluble-to-soluble NSPSs than those of medium or good quality, confirming that NSPSs, especially hemicelluloses, play an important role in final bread quality. This result is in accordance with the studies performed by Stępniewska et al. (2018), who showed a possible prediction of dough and bread quality using a curve swelling test. The results of the curve swelling test are decisively influenced by the presence and composition of hemicelluloses [20].

Arabinoxylans are the most abundant hemicelluloses in rye [51]. Their structural properties and branching of the xylose backbone with arabinose influence their water-binding capacity and enzymatic degradation. The insoluble, nonextractable (in water) arabinoxylans have a negative influence on dough properties compared to their water-extractable counterparts. Consequently, their influence on starch granules was analyzed.

By quantification of NSPSs in the starch phase, a coherence between baking quality and pentosan and glucan contents could be determined, linking a high amount of pentosans and glucans adhering to starch granules with poor baking quality. This suggests that hemicelluloses interact with starch granules in a way that negatively affects dough formation and stability. It is known that proteins can form layers around starch granules, and it is also known that proteins and hemicelluloses (e.g., arabinoxylans) can form complexes [48]. Whether the hemicelluloses interact with starch granules directly or via interactions with proteins is currently being studied.

Although protein content could not be correlated with baking quality directly, a low conversion enthalpy in the gel phase, despite higher protein content, illustrated an inadequate denaturation process; this caused a lack of free water necessary for starch gelatinization. This might be evoked by the interactions of the proteins with hemicelluloses, hindering the denaturation process.

Additionally, the high residual enthalpy of partially gelatinized starch with excess water, measured for the samples of poor baking quality, indicate a lack of water for the process (caused by competition with other components such as hemicelluloses), hindering starch gelatinization due to the limited water supply, although the starch is, in principle, accessible to water.

It can be assumed that a complex formation between hemicelluloses and proteins interferes with starch gelatinization during dough formation and, thus, leads to quality defects in bread rye. The results suggest two possible scenarios regarding poor baking quality: (1) The starch is, in principle, accessible to water, but the free water needed for gelatinization is permanently absorbed by hemicelluloses and/or hemicellulose–protein complexes. These complexes might also inhibit protein denaturation and, thereby, inhibit the release of free water. (2) The starch is not accessible to water due to the accumulation of proteins, hemicelluloses, and/or hemicellulose–protein complexes on the surface of the starch granules. When comparing protein crosslinks that are artificially formed with transglutaminase and the (plant) complexes endogenously formed with ferulic acid, there might be a different outcome. While the enzymatically induced complexes of molecular weight seem to lead to improved baking quality [43], the ferulic-acid-connected heterocrosslinks might hinder water accessibility and baking quality [50]. Precise mechanisms by which these three components (starch, hemicelluloses, and proteins) might interact still need to be studied. The detection and elucidation of the type of complexes could possibly identify biomarkers for poor bread quality. In order to prove this thesis, however, further studies must be carried out.

## Figures and Tables

**Table 1:** Selected rye flours and their main characteristics according to the standard baking test.

Sample No.	Cultivar	FN (s) ^1^	Gelatinization Temperature (°C)	Gelatinization Maximum (ΔE)	Points/Diff. of Sensory Assessment ^2^	Baking Quality Class
**1**	Baro	164	66.9	432	17/3	good
**2**	Brasetto	271	74.0	807	17/1	good
**3**	Minello	153	68.1	514	14/4	medium
**4**	Palazzo	207	70.3	836	14/2	medium
**5**	Palazzo	185	68.8	786	12/4	poor
**6**	Palazzo	251	72.1	875	10/4	poor

^1^ FN = falling number (c.f. Section 2.2.1); ^2^ points/diff. = result of the baking test after 24 /120 h (c.f. Section 2.2.3).

## Supplementary Materials

The following are available online at https://www.mdpi.com/2304-8158/9/12/1900/s1, Figure S1: Xylose standard curve with polynomial fit (degree = 2) for different xylose concentrations, Figure S2: Starch content of the selected samples, depending on the baking quality classes from ‘good’ to ‘medium’ to ‘poor’ and the corresponding falling number (FN) within the individual classes, Figure S3: Protein content of the selected samples, depending on the baking quality classes from ‘good’ to ‘medium’ to ‘poor’ and the corresponding falling number (FN) within the individual classes, Table S1: Sensory characteristics and scoring of rye bread products after the standard baking test for the definition of a baking quality score, Table S2: Results from the quantitative analysis of the chemical composition from six selected rye flours.
