# Supplementary material for: Rye Bread Defects: Analysis of Composition and Further Influence Factors as Determinants of Dry-Baking
Source: Foods. 2020 Dec 19;9(12):1900. doi: 10.3390/foods9121900 (PMC7765839; doi:10.3390/foods9121900)
Supplement: Supplementary file 1 [file foods-09-01900-s001.zip › supplementary material/foods-1025252_supplementary material.docx]

Supplementary Material

**Table S1.** Sensory characteristics and scoring of rye bread products after the standard baking test for the definition of a baking quality score.

| Characteristics | Description | max. score |
| --- | --- | --- |
| 1) Volume, shape | volume impression, curvature, shoulder formation, edge cracks, side surface | 2 |
| 2) crust | condition of surface and bottom/crust color/crust thickness | 2 |
| 3) crumb | loosening, poring, crumbling, compacting or grinding | 4 |
|  | crumb elasticity (pressure test), tactile impression, stickiness | 3 |
|  | structural impression, cuttability, bite/chewability, solubility | 4 |
| 4) Smell and taste | purity, aroma impression, taste impression, acidity | 4 |
| Total score | | 19 |


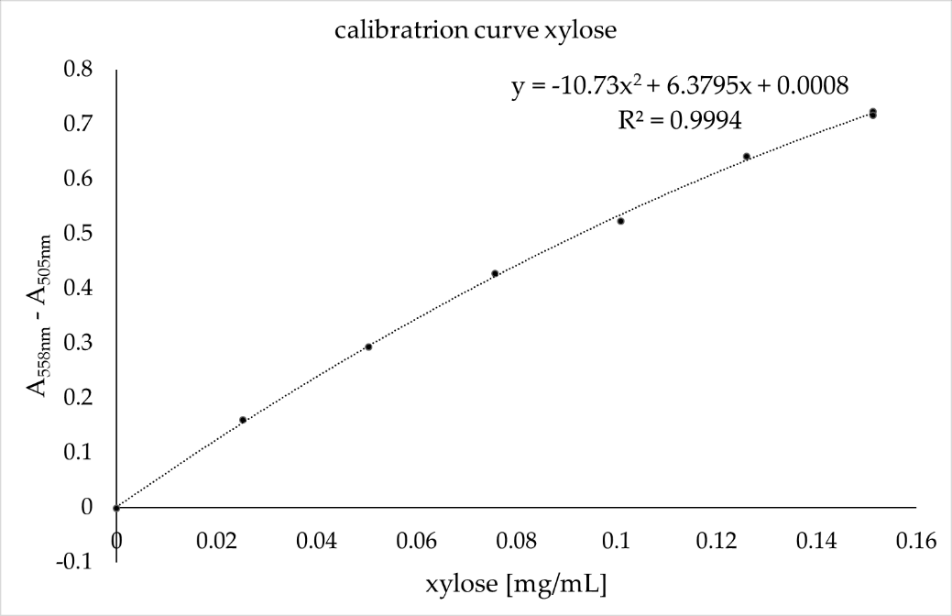


**Figure S1.** Xylose standard curve with polynomial fit (degree = 2) for different xylose concentrations.


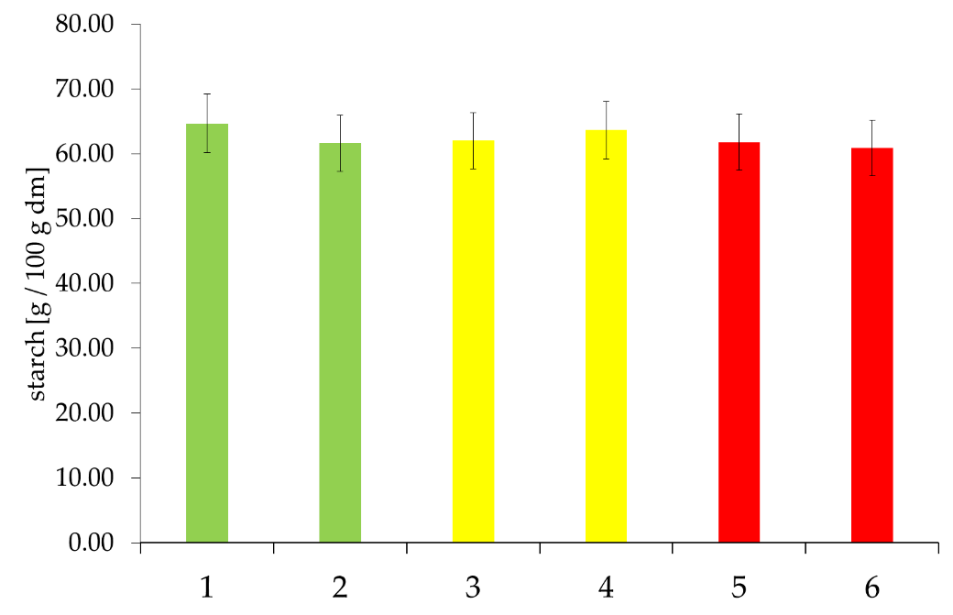


**Figure S2.** Starch content of the selected samples, depending on the baking quality classes from ‘good’ to ‘medium’ to ‘poor’ and the corresponding falling number (FN) within the individual classes (here: first samples always assigned to the lower FN and second samples with the higher FN). Sample description: see Table 1. Bars represent the mean value of two replicates; error bars represent the confidence interval.


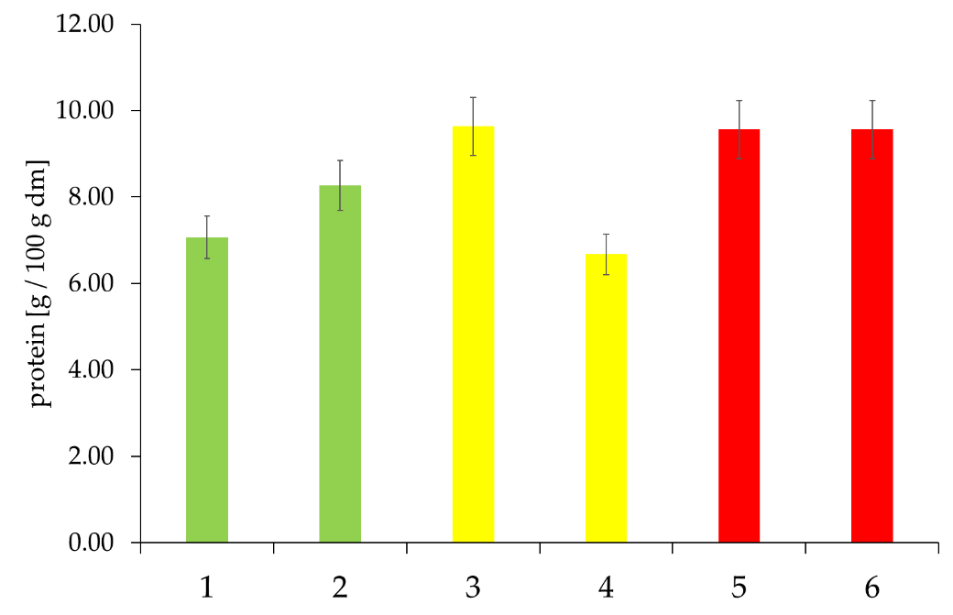


**Figure S3.** Protein content of the selected samples, depending on the baking quality classes from ‘good’ to ‘medium’ to ‘poor’ and the corresponding falling number (FN) within the individual classes (here: first samples always assigned to the lower FN and second samples with the higher FN). Sample description: see Table 1. Bars represent the mean value of two replicates; error bars represent the confidence interval.

**Table S2.** Results from the quantitative analysis of the chemical composition from six selected rye flours.

|  |  | 1 | 2 | 3 | 4 | 5 | 6 |
| --- | --- | --- | --- | --- | --- | --- | --- |
| Phase | Parameter | Baro | Brasetto | Minello | Palazzo | Palazzo | Palazzo |
| Flour | FN [s] | 164 | 271 | 153 | 207 | 185 | 251 |
|  | Amylose/Amylopectin | 0.373 | 0.366 | 0.341 | 0.354 | 0.343 | 0.342 |
|  | insol./sol. NSPS | 1.789 | 1.693 | 1.765 | 1.734 | 2.284 | 1.930 |
|  | sol. Protein [%]^a^ | 33.95 | 26.81 | 23.64 | 25.37 | 24.00 | 22.47 |
| Starch | Protein I/II^b^ [% in dm] | 0.41/0.62 | 0.37/1.1 | 1.08/1.6 | 0.29/0.99 | 0.38/1.0 | 0.44/1.51 |
|  | Pentosans [% in dm] | 0.022 | 0.034 | 0.099 | 0.166 | 0.133 | 0.199 |
|  | Glucans [% in dm] | - | 0.046 | 0.066 | 0.166 | 0.100 | 0.287 |
|  | res. Enthalpy I/II^b^  [J/g dm]^c^ | 0.26/0.09 | 0.30/0.15 | 0.41/0.26 | 0.35/0.11 | 0.23/1.80 | 2.20/1.86 |
| Gel | Protein [% in dm] | 17.84 | 16.00 | 19.49 | 15.45 | 22.48 | 20.38 |
|  | NSPS [% in dm] | 9.21 | 6.40 | 14.68 | 12.83 | 15.23 | 14.24 |
|  | conv. Enthalpy  [J/g protein] | 25.4 | 29.1 | 20.8 | 29.0 | 6.3 | 13.0 |

^a^ based on total protein content

^b^ I = starch phase I; II = starch phase II

^c^ after partial gelatinization
