# Supplementary figures and images for: Rye Bread Defects: Analysis of Composition and Further Influence Factors as Determinants of Dry-Baking
Source: Foods. 2020 Dec 19;9(12):1900. doi: 10.3390/foods9121900 (PMC7765839; doi:10.3390/foods9121900)

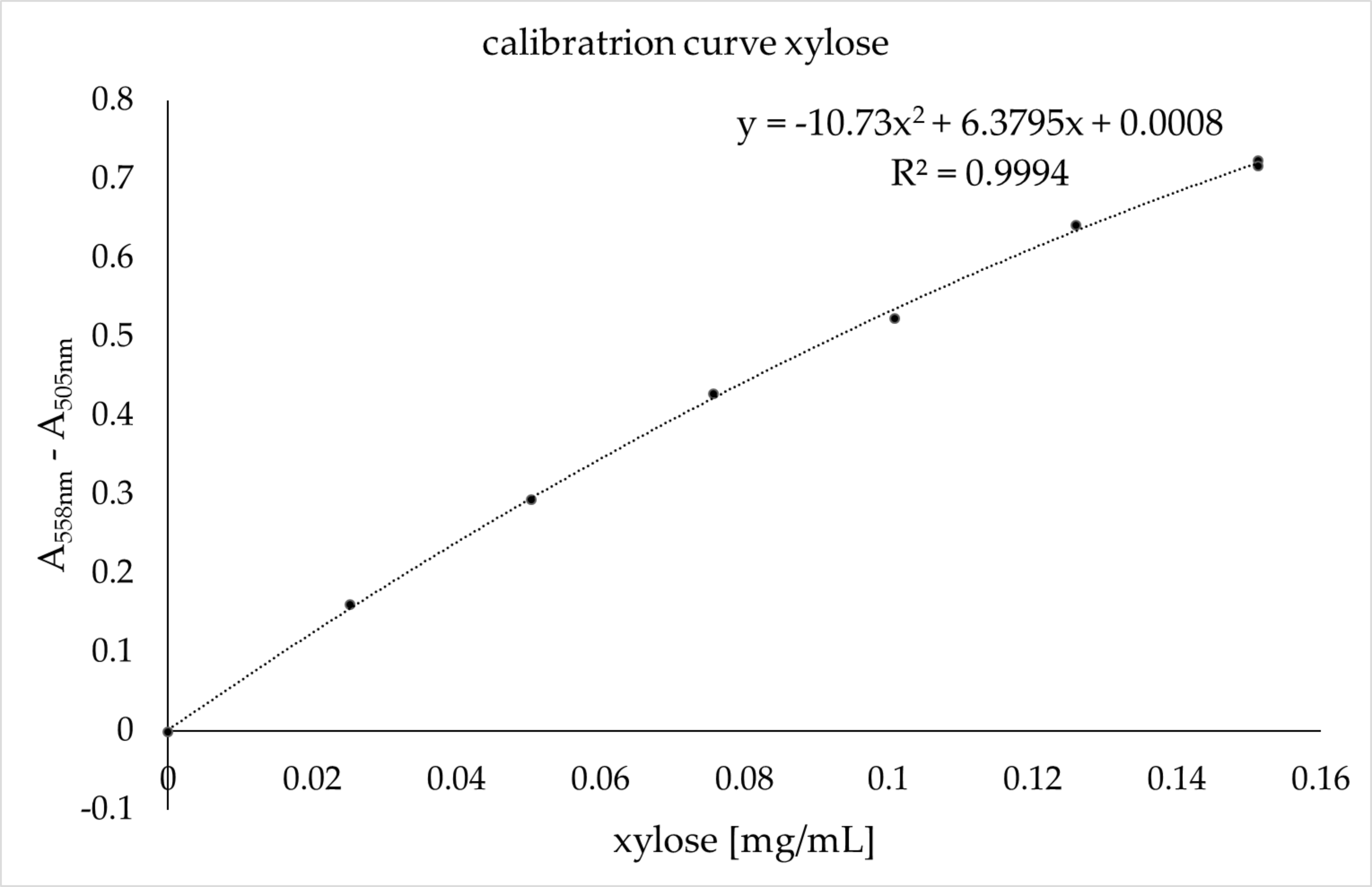

Supplement: Supplementary file 1 [file foods-09-01900-s001.zip › supplementary material/calibration curve xylose.png]

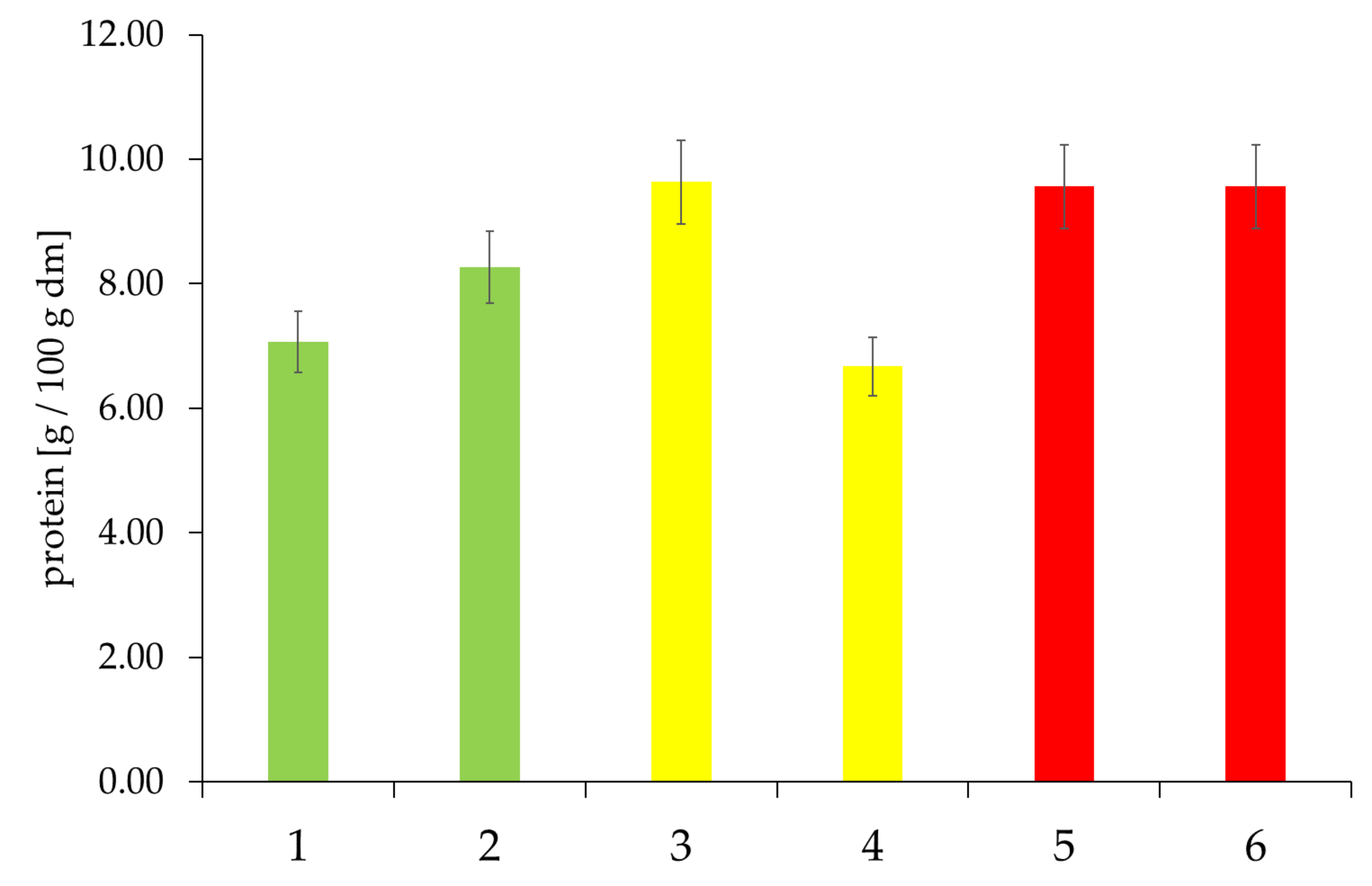

Supplement: Supplementary file 1 [file foods-09-01900-s001.zip › supplementary material/protein content_flour.png]

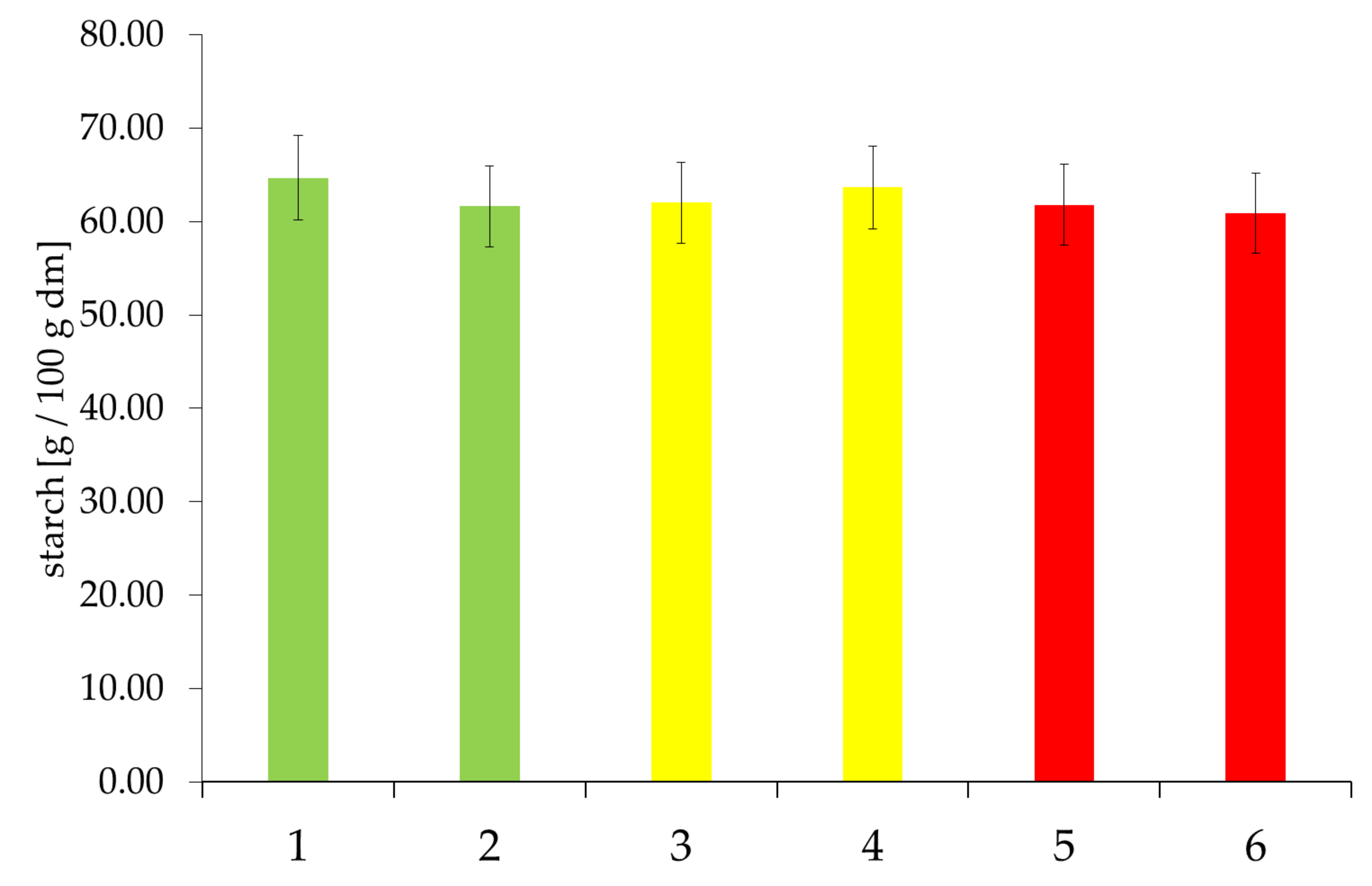

Supplement: Supplementary file 1 [file foods-09-01900-s001.zip › supplementary material/starch content_flour.png]

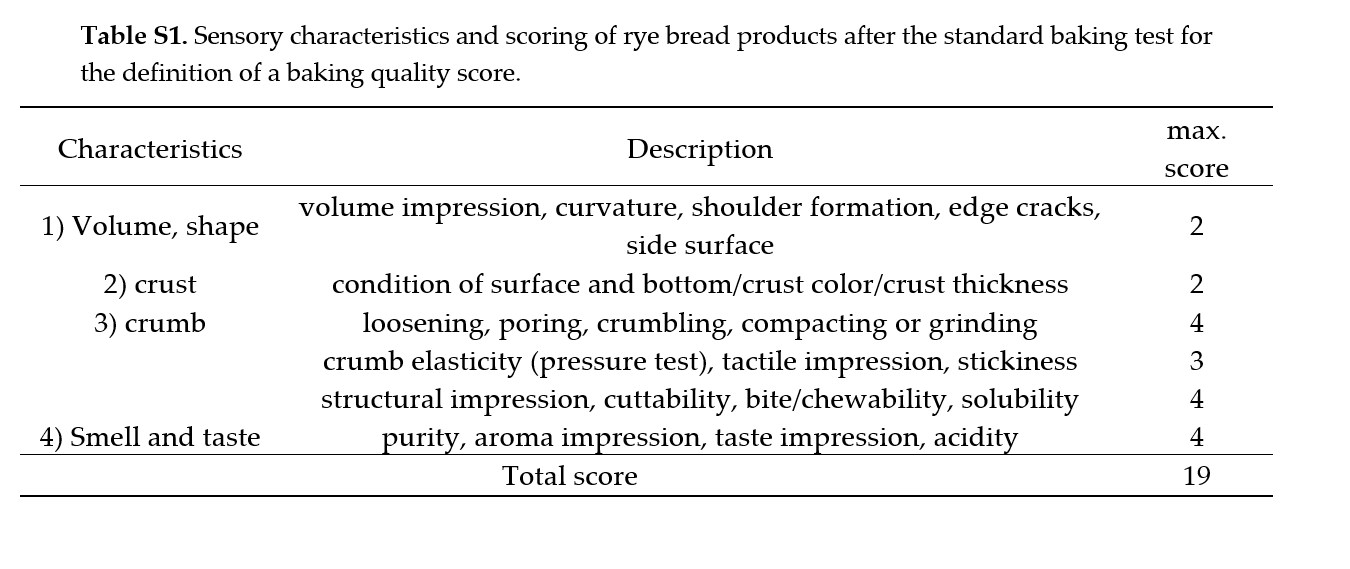

Supplement: Supplementary file 1 [file foods-09-01900-s001.zip › supplementary material/table S1_sensory characteristics and scoring.PNG]

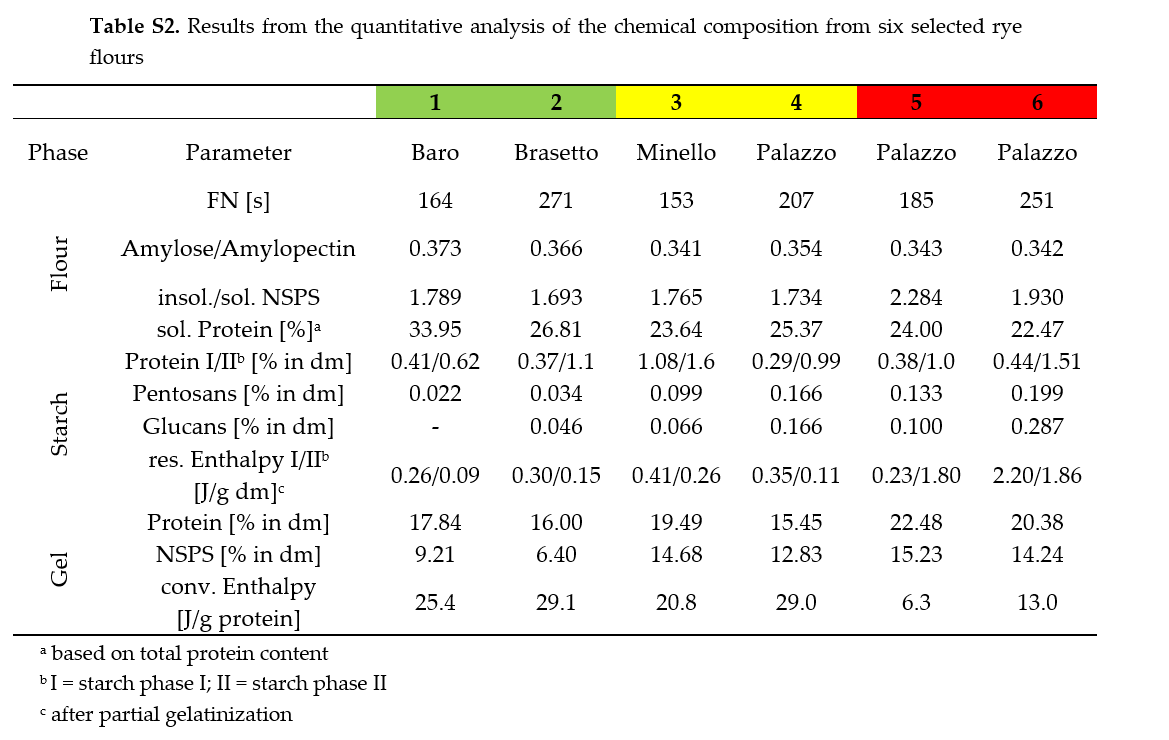

Supplement: Supplementary file 1 [file foods-09-01900-s001.zip › supplementary material/table S2_results quantitative analysis.PNG]
